# Association between variation of circulating 25-OH vitamin D and methylation of secreted frizzled-related protein 2 in colorectal cancer

**DOI:** 10.1186/s13148-020-00875-9

**Published:** 2020-06-09

**Authors:** Hatim Boughanem, Amanda Cabrera-Mulero, Pablo Hernández-Alonso, Mercedes Clemente-Postigo, Felipe F. Casanueva, Francisco José Tinahones, Sonsoles Morcillo, Ana B. Crujeiras, Manuel Macias-Gonzalez

**Affiliations:** 1 Biomedical Research Institute of Malaga (IBIMA), Faculty of Science, University of Malaga, 29010 Málaga, Spain; 2 Deparment of Endocrinology and Nutrition, Virgen de la Victoria University Hospital, Institute of Biomedical Research in Malaga (IBIMA) and University of Malaga, Malaga, Spain; 3 CIBER Physiopathology of Obesity and Nutrition (CIBERobn), Institute of Health Carlos III, Madrid, Spain; 4 Human Nutrition Unit, Faculty of Medicine and Health Sciences, Sant Joan Hospital, Institut d’Investigació Sanitària Pere Virgili, Rovira i Virgili University, 43201 Reus, Spain; 5 Department of Cell Biology, Physiology and Immunology, Instituto Maimónides de Investigación Biomédica de Córdoba (IMIBIC)-Reina Sofia University Hospital, University of Cordoba, Córdoba, Spain; 6 Epigenomics in Endocrinology and Nutrition Group, Instituto de Investigación Sanitaria (IDIS), Complejo Hospitalario Universitario de Santiago (CHUS/SERGAS), Santiago de Compostela, Spain

**Keywords:** *SFRP2* methylation, 25-OH vitamin D, Colorectal cancer, Neoadjuvant treatment

## Abstract

**Backgrounds:**

Colorectal cancer (CRC) results from the accumulation of epigenetic and genetic changes in colon cells during neoplasic transformation, which the activation of Wingless (Wnt) signaling pathway is a common mechanism for CRC initiation. The Wnt pathway is mainly regulated by Wnt antagonists, as secreted frizzled-related protein (SFRP) family. Indeed, SFRP2 is proposed as a noninvasive biomarker for CRC diagnosis. Vitamin D also antagonizes Wnt signaling in colon cancers cells. Several studies showed that vitamin D was able to alter DNA methylation, although this mechanism is not yet clear. Therefore, the aim of this study was to find an association between circulating 25-OH vitamin D (30th percentile of vitamin D) and the *SFRP2* methylation.

**Methods:**

A total of 67 CRC patients were included in the study. These patients were subdivided into two groups based on their 30th percentile vitamin D (20 patients were below, and 47 participants were above the 30th percentile of vitamin D). We investigated the *SFRP2* methylation in peripheral blood mononuclear cells (PBMCs), visceral adipose tissue (VAT), CRC tumor tissue, and adjacent tumor-free area. We also determined the relationship between *SFRP2* methylation and methylation of carcinogenic and adipogenic genes. Finally, we tested the effect of vitamin D on the *SFRP2* methylation in human colorectal carcinoma cell lines 116 (HCT116) and studied the association of neoadjuvant therapy under the 30th percentile vitamin D with *SFRP2* promoter methylation.

**Results:**

*SFRP2* methylation in tumor area was decreased in patients who had higher levels of vitamin D. *SFRP2* promoter methylation was positively correlated in tumor area with insulin and homeostasis model assessment of insulin resistance (HOMA-IR) but negatively correlated with HDL-c. *SFRP2* methylation was also correlated with T cell lymphoma invasion and metastasis 1 (*TIAM1*) methylation in tumor area and CCAAT/enhancer-binding protein alpha (*C/EBPα*) in VAT. Treatment with vitamin D did not affect *SFRP2* methylation in HCT116 cell line. Finally, neoadjuvant treatment was correlated with higher circulating 25-OH vitamin D and *SFRP2* methylation under linear regression model.

**Conclusion:**

Our results showed that higher circulating vitamin D is associated with low *SFRP2* promoter methylation. Therefore, our results could suggest that vitamin D may have an epigenetic effect on DNA methylation. Finally, higher vitamin D could contribute to an improvement response to neoadjuvant treatment.

## Background

Colorectal cancer (CRC) is one of the most common cancers worldwide. Combined, in both sexes, CRC is the third most commonly diagnosed cancer and one of the leading causes of cancer-related mortality [1]. The mechanism underlying CRC carcinogenesis remains subject of intense research. It is well known that genetic and epigenetic alterations in cell crypt foci lead to activation of Wingless (Wnt) signaling pathway [2]. Indeed, aberrant activation of Wnt signaling is a hallmark of CRC and is therefore one of the most investigated target for preventive and therapeutic intervention [3]. Uninterrupted activation of the Wnt pathway is often caused by alterations in any of their components, either by mutational or epigenetic changes and therefore leads to uncontrolled cancer cell proliferation and differentiation [4, 5].

The Wnt signaling pathway is mainly regulated by Wnt antagonists, which members of secreted frizzled-related protein (SFRP types 1 to 5) family were the first Wnt antagonists to be identified [6]. SFRP members are extracellular secreted glycoproteins that directly bind to Wnt proteins and functionally inhibit its mechanism of action. Ergo, the main role of SFRPs is focused on preventing Wnt action and decreasing Wnt signaling intensity [7]. This inactivation of Wnt signaling is often caused by *SFRPs* promoter hypermethylation [8, 9]. Specifically, *SFRP2* hypermethylation (and consequent loss of *SFRP2* expression) has widely been documented in several cancer types, such as gastric cancer [10], osteosarcoma [11], and breast cancer [12]. Furthermore, several studies consider abnormal methylation of *SFRP2* as a noninvasive diagnostic and prognostic biomarker of CRC [13–15], suggesting that SFRP2 might be implicated in cancer development, but also as a tumor suppressor gene. However, more studies are required to understand the role of SFRP2 and its molecular signaling pathways in CRC. The interaction of SFRP2 with other pathway components should lead to identify novel therapeutic targets.

On the other hand, a series of studies reported that vitamin D also antagonizes Wnt signaling in colon cancers cells [16, 17]. Vitamin D is well known as an essential component for calcium and phosphorus absorption and bone formation, but it is also proposed as a pleiotropic hormone with many anti-cancer effects, such as inhibition of cancer cell proliferation and migration [18]. There are also evidences that vitamin D is able to alter DNA methylation, although this mechanism is not yet clear [19]. In any case, this fact is relatively novel, and more investigations are coming to elucidate the role of vitamin D on DNA methylation.

For instance, a study found that circulating 25-OH vitamin D was negatively associated with adenomatous polyposis coli (*APC*) promoter methylation and positively associated with methylation of genomic long interspersed nuclear element-1 (*LINE1*) in human rectal mucosa [20]. Another study showed that vitamin D intake was correlated with decreased promoter methylation of dickkopf1 (*DKK1*) and *Wnt5A* (both considered Wnt antagonists) in CRC tumors [21]. Therefore, it could be interesting to verify whether vitamin D may regulate DNA methylation as epigenetic modifier with well-known modes of action.

In addition, vitamin D improves treatment of many types of cancer, by potentiating the anti-tumoral activity of chemotherapeutic agents and enhancing the cytotoxic effect of ionizing irradiation [22]. Recently, a new randomized clinical trial demonstrated that high doses of vitamin D slowed the growth of colorectal cancer in patients with started chemotherapy. The idea that vitamin D could be in synergy with chemotherapy treatment is very promising and suggests an improved outcome for CRC patients. However, more studies are needed to determine how vitamin D supplementation dose could improve outcome in CRC patients who take neoadjuvant treatment [23].

Here, we hypothesized that low circulating 25-OH vitamin D could be associated with promoter methylation of *SFRP2* in CRC patients, and this state could be important in treatment strategy. Therefore, the aim of this study was to investigate the association between circulating 25-OH vitamin D and the *SFRP2* methylation. We also studied the relationship between *SFRP2* methylation and methylation of carcinogenic and adipogenic genes to evaluate a possible relationship between vitamin D and DNA methylation in CRC patients.

## Results

### Anthropometric and biochemical variables of CRC patients, according to the 30th percentile of vitamin D

Anthropometric and biochemical variables of CRC patients who are below the 30th percentile of vitamin D (< 30th CRC group) (*N* = 20) and CRC patients who are above the 30th percentile of vitamin D (> 30th CRC group) (N = 47) are shown in Table 1. The 30th percentile of vitamin D was calculated taking place our CRC cohort. There were not significant differences between age, gender, anthropometric variables, glucose metabolism variables, and lipid profile variables between both groups. In contrast, as expected, vitamin D levels were significantly decreased in the < 30th group than in the > 30th group (*p* < 0.05), which the mean value of 25-OH vitamin D was 16.00 ± 4.73 ng/ml and 37.11 ± 9.41 ng/ml, respectively.

**Table 1:** Clinical, anthropometric, and biochemical variables of CRC patients, according to their 30th percentile of vitamin D

Variables	< 30th percentile of 25-OH vitamin D	> 30th percentile of 25-OH vitamin D	*p* value
*N*	20	47	0.347
Age (years)	68.5 ± 9.42	66.06 ± 9.97	0.351
Male/female (%)	12/8, 60/40	34/13, 72/28	0.319
BMI (kg/m^2^)	27.14 ± 4.12	27.46 ± 4.39	0.779
Waist circumference (cm)	96.20 ± 14.28	99.12 ± 13.73	0.612
Glucose (mg/dl)	126.21 ± 53.89	121.08 ± 40.39	0.711
Insulin (μUI/ml)	5.52 ± 3.68	6.41 ± 5.99	0.481
HOMA-IR	1.58 ± 1.28	2.19 ± 2.37	0.219
Triglycerides (mg/dl)	206.31 ± 112.00	152.98 ± 63.78	0.063
Total cholesterol (mg/dl)	161.89 ± 28.57	174.95 ± 48.30	0.180
HDL-c (mg/dl)	35.58 ± 19.24	42.11 ± 11.98	0.182
LDL-c (mg/dl)	97.74 ± 23.47	105.08 ± 40.06	0.359
Corrected calcium (mg/dl)	9.06 ± 0.49	8.95 ± 0.43	0.410
Alkaline phosphatase(U/l)	74.26 ± 29.01	64.79 ± 29.26	0.239

Data are expressed as mean ± standard deviations or percentage. Asterisk indicates significant difference between the < 30th and > 30th percentile of vitamin D according to Welch’s two sample test. Chi squared test was used for variables expressed as percentage (*p* < 0.05)

*CRC* colorectal cancer, *BMI* body mass index, *HOMA-IR* homeostasis model assessment of insulin resistance, *HDL-c* high density lipoprotein cholesterol, *LDL-c* low density lipoprotein cholesterol

### *SFRP2* promoter methylation in PBMCs, VAT, CRC tumor area, and CRC tumor-free area according to the 30th percentile of vitamin D

**Fig. 1:**
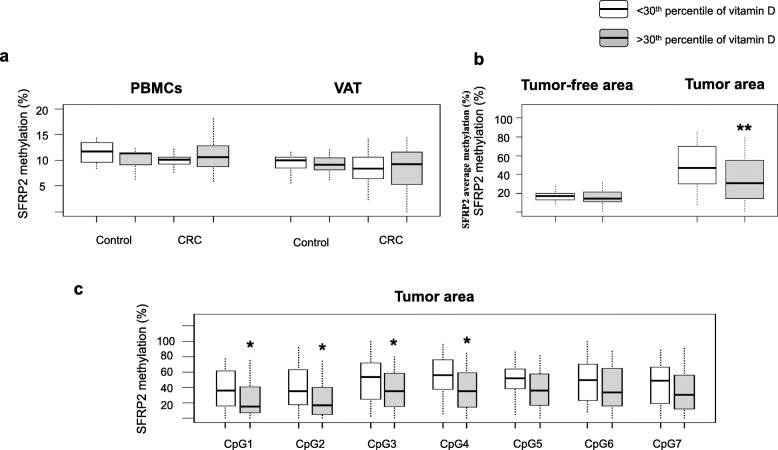
Methylation analyses of *SFRP2* promoter in different tissues in non-CRC subjects and CRC patients, according to the 30th percentile of 25-OH vitamin D. **a** Methylation average status for the *SFRP2* promoter in PBMCs (*N* = 11; *N* = 52) and VAT (*N* = 53; *N* = 50) from non-CRC and CRC patients, respectively. **b** Methylation average status for the *SFRP2* promoter in CRC tumor area (*N* = 60) and CRC tumor-free area (*N* = 55) from CRC patients. **c** Methylation analyses of seven CpG islands of the *SFRP2* promoter in tumor area from CRC patients (*N* = 60). The results are given as methylation average ± standard deviation. Significant differences between the means of the different groups of subjects were performed according to the Welch’s two sample test (*p < 0.05, **p < 0.01). Abbreviations: SFRP2, Secreted frizzled-related protein type 2; CRC, Colorectal cancer; PBMCs, peripheral blood mononuclear cells; VAT, Visceral adipose tissue

To check whether circulating 25-OH vitamin D levels may be related to the promoter methylation of *SFRP2* gene, we studied the methylation status of *SFRP2* in different biological tissues from CRC patients according to their 30th percentile of vitamin D (Fig. 1). Firstly, we compared the methylation average of *SFRP2* in peripheral blood mononuclear cells (PBMCs) and visceral adipose tissue (VAT) from non-CRC participants and CRC patients classified according to their 30th percentile of vitamin D. Our results showed that not significant differences were observed in *SFRP2* promoter methylation between < 30th and > 30th groups in PBMCs and VAT from non-CRC subjects (Fig. 1a). The same results were observed in CRC patients (Fig. 1a). When we compared CRC tumor tissue, *SFRP2* promoter methylation was significantly decreased in the > 30th CRC group than in the < 30th CRC group (*p* < 0.01) (Fig. 1b). However, we did not find significant differences in *SFRP2* methylation from adjacent tumor-free area between both groups (Fig. 1b).

Finally, when deepened in the study of the seven CpG islands of *SFRP2* promoter of tumor colon area, the methylation of CpG sites 1, 2, 3, and 4 was significantly decreased in the > 30th group, when compared to the < 30th group (*p* < 0.05) (Fig. 1c). Results with treatment with vitamin D in HCT116 cells did not affect *SFRP2* methylation (Figure S3).

### Metabolic variables analysis: correlation between *SFRP2* and anthropometric and biochemical variables

**Fig. 2:**
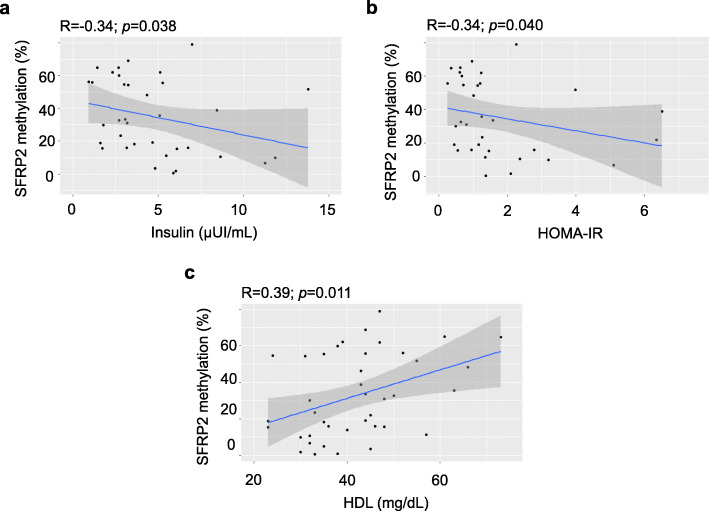
Correlation between *SFRP2* promoter methylation and metabolic parameters in CRC patients above the 25-OH vitamin D 30th percentile. Correlation between *SFRP2* promoter methylation in tumor area (*N* = 60) from CRC patients and metabolic variable, in CRC patients above the 30th percentile of 25-OH vitamin D. Pearson’s correlation was performed to determine correlation between methylation analyses and variables. Abbreviations: HOMA-IR, Homeostasis model assessment of insulin resistance; HDL-c, High density lipoprotein cholesterol; SFRP2, Secreted frizzled-related protein type 2

To verify the relationship between *SFRP2* methylation and biochemical parameters, we performed Pearson’s correlation analysis. We found that *SFRP2* promoter methylation was positively correlated in tumor area with insulin and HOMA-IR but negatively correlated with HDL-c (Fig. 2). The promoter methylation of *SFRP2* was also positively correlated in adjacent tumor-free area with insulin (Table S1). Table S1 shows the correlation between *SFRP2* methylation in different tissues and anthropometric and biochemical variables.

### Tumor methylation analysis: correlation between *SFRP2* methylation and genes related to colorectal carcinogenesis

**Fig. 3:**
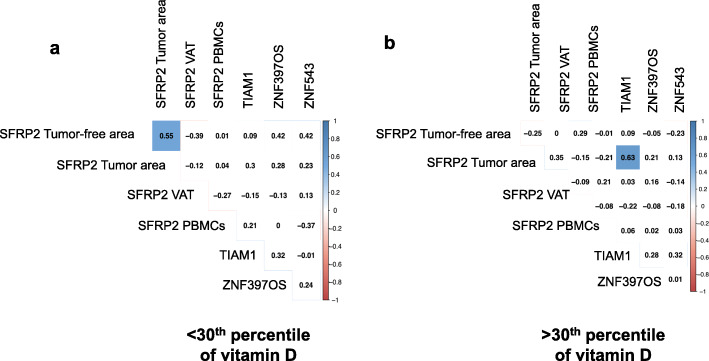
Tumor methylation analysis: correlation between *SFRP2* promoter methylation status and promoter methylation of genes related to colorectal carcinogenesis in tumor tissue from CRC patients, according to 30th percentile of vitamin D. Correlation between *SFRP2* promoter methylation analyses in PBMCs (*N* = 52), tumor area (*N* = 60), tumor-free area (*N* = 55), and VAT (*N* = 50) from CRC patients and promoter methylation of *TIAM1*, *ZNF397OS*, and *ZNF543* in tumor area, according to the **a** < 30th percentile and **b** > 30th percentile. Pearson’s correlation was performed to determine correlation between methylation analyses. Data are expressed as correlation coefficient. Colored squares indicate significant difference between methylated genes according to the Pearson’s analysis (*p* < 0.05). Abbreviations: CRC, Colorectal cancer; SFRP2, Secreted frizzled-related protein type 2; PBMCs, peripheral blood mononuclear cells; VAT, Visceral adipose tissue; TIAM1, T cell lymphoma invasion and metastasis 1; ZNF397OS, Zinc Finger Protein 397; ZNF543, Zinc Finger Protein 543

Previously, our group identified an episignature of human colorectal cancer associated with obesity and demonstrated that methylation of T cell lymphoma invasion and metastasis 1 (*TIAM1*), Zinc finger protein type 397OS (*ZNF397OS*), and 543 (*ZNF543*) genes represented the top genes scoring, associated with colorectal cancer and an increased body mass index (BMI) in two independent cohorts [24]. For that, we studied the association between promoter methylation of these genes and *SFRP2* methylation in different tissues, according to the 30th percentile of vitamin D (Fig. 3).

In CRC patients who are below of 30th percentile of vitamin D, *SFRP2* methylation did not correlate with methylation of colorectal carcinogenesis genes, although *SFRP2* methylation in tumor area was positively correlated with *SFRP2* methylation in tumor-free area (*p* < 0.05) (Fig. 3a). However, in > 30th CRC group, *SFRP2* methylation was positively correlated with promoter methylation of *TIAM1* in tumor area (*p* < 0.05) (Fig. 3b).

### Visceral adipose tissue methylation analysis: correlation between *SFRP2* methylation and genes related to adipogenesis and inflammation processes

**Fig. 4:**
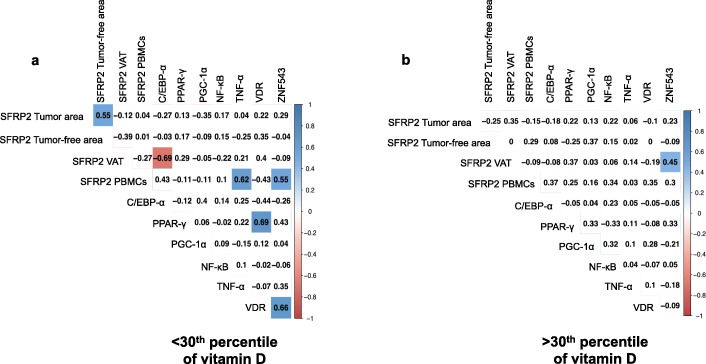
Visceral adipose tissue methylation analysis: correlation between *SFRP2* promoter methylation status and methylation of genes related to adipogenesis and inflammation in visceral adipose tissue from CRC patients, according to the 30th percentile of vitamin D. Correlation between *SFRP2* promoter methylation analyses in PBMCs (*N* = 52), tumor area (*N* = 60), tumor-free area (*N* = 55), and VAT (*N* = 50) from CRC patients and *C/EBPα*, *PPARγ*, *PGC1α*, *NFκB*, *TNFα*, *VDR*, and *ZNF543* promoter methylation in tumor area, according to the **a** < 30th percentile and **b** > 30th percentile of vitamin D. Pearson’s correlation was performed to determine correlation between methylated genes. Data are expressed as correlation coefficient. Colored square indicates significant difference between methylated genes according to Pearson’s analysis (*p* < 0.05). Abbreviations: CRC, Colorectal cancer; SFRP2, Secreted frizzled-related protein type 2; PBMCs, peripheral blood mononuclear cells; VAT, Visceral adipose tissue; C/EBPα, CAATT/Enhancer Binding Protein type α; PPARγ, Peroxisome proliferator activator receptor type γ; PGC1α, PPARγ coactivator type 1α; NFκB, nuclear factor κ B; TNFα, Tumor necrosis factor type α; VDR, Vitamin D receptor; ZNF543, Zinc finger protein type 543

In order to determine the role of *SFRP2* methylation in VAT and CRC, we studied the relationship between key genes in adipose tissue, implicated in adipogenesis as CCAAT/enhancer-binding protein alpha (*C/EBPα*), peroxisome proliferator activated receptor gamma (*PPARγ*), peroxisome proliferator activated receptor gamma coactivator 1 alpha (*PGC1α*), and inflammatory processes, as nuclear factor kappa B (*NFκB*) and tumor necrosis factor alpha (*TNFα*) and vitamin D receptor (*VDR*) (Fig. 4). In the < 30th CRC group, *SFRP2* methylation was negatively associated with *C/EBPα* promoter methylation in VAT (*p* < 0.05). Moreover, *VDR* methylation was also positively correlated with *PPARγ* and *ZNF543* methylation in adipose tissue (*p* < 0.05) (Fig. 4a). Additionally, in CRC > 30th CRC group, *SFRP2* methylation was only correlated with promoter methylation of *ZNF543* in adipose tissue (*p* < 0.05) (Fig. 4b).

### *SFRP2* methylation and high vitamin D levels are associated with neoadjuvant treatment in CRC

Our group demonstrated that the *SFRP2* methylation was associated with the neoadjuvant treatment in CRC. The methylation average of *SFRP2* was lower in treated patients than in the non-treated patients [15]. Therefore, we studied the association between circulating vitamin D and *SFRP2* methylation taking neoadjuvant treatment as independent variable. A linear regression analysis was performed, in the presence of other potential confounder variables such as age, gender, and BMI (Table 2). Interestingly, taking *SFRP2* methylation as dependent variable, neoadjuvant treatment reached a strong significance in the > 30th CRC group and was the main variables. This model was able to explain up to 24% of the variability of the *SFRP2* methylation.

**Table 2:** Multivariate linear regression analysis as predictor of response of neoadjuvant treatment using *SFRP2* methylation promoter

Variables	< 30th percentile of vitamin D; *R*^2^ = 0.23; *R*^2#^ = 0.01; *p* = 0.412		> 30th percentile of vitamin D; *R*^2^ = 0.32; *R*^2#^ = 0.24; *p* = 0.009*	
	β (SE)	95% CI	β (SE)	95% CI
Gender	2.87 (11.28)	(− 21.32 to 27.07)	11.87 (7.55)	(− 3.48 to 27.21)
Age (years)	0.09 (0.66)	(− 1.31 to 1.50)	− 0.63 (0.37)	(− 1.40 to 0.12)
BMI	0.82 (1.46)	(− 2.31 to 3.95)	− 0.70 (0.85)	(− 2.44 to 1.04)
Neoadjuvant treatment	− 9.43 (4.68)	(− 19.48 to 0.61)	− 8.15 (2.58)**	**(**− **13.40 to (**− **2.90))**

The multivariate linear regression analyses were conducted using *SFRP2* promoter methylation in tumor area as dependent variable, and age, gender, BMI, and treatment after surgery in tumor area as independent parameters, according to the 30th percentile of vitamin D (# adjusted). Asterisk indicates significant correlation (*p < 0.05, **p < 0.01)

*SFRP2* secreted frizzled-related protein type 2

## Discussion

Several epidemiological and clinical trial studies showed the beneficial effects of vitamin D in colorectal cancer prevention and chemotherapy [25]. A recent study found that higher levels of circulating vitamin D were associated with a lower risk for CRC, which optimal concentrations of 25-OH vitamin D for reducing risk of CRC was between 30 and 40 ng/ml, as observed by our study [26]. Although previous studies have suggested a possible link but were inconclusive, inconsistent, or contradictory, and new large-scale clinical trials are needed. Therefore, diverse mechanisms have been proposed to explain the benefits of vitamin D in CRC.

At the epigenetic level, vitamin D acts by binding to VDR and affects transcriptions of key tumor suppressor genes [27]. The epigenetic effects of vitamin D are associated with hypomethylation of key genes implicated in carcinogenesis such as *p21*, phosphatase and tensin homolog (*PTEN*), Cadherin 1 (*CDH1*), and a number of tumor suppressor genes [28]. The evidence that vitamin D could modify DNA methylation is relatively novel (but may be attributed to the negative regulation of DNMT1 activity) [29]. A recent study found that vitamin D intake was correlated with promoter methylation of *DKK1* (Wnt antagonist as SFRPs) in CRC patients [30]. However, the main effect of vitamin D on DNA methylation is not clear yet.

According to these results, in our study, we found for the first time that higher circulating vitamin D is associated with low *SFRP2* promoter methylation. The > 30th CRC group had significantly decreased *SFRP2* methylation in tumor area (but not in PBMCs, VAT, and tumor-free area) in comparison with the < 30th CRC group. The first four CpG sites were different in both groups.

As far as we understand, SFRP2 functions as a tumor suppressor gene by binding to Wnt protein as antagonist and inhibiting Wnt signaling pathway [31]. Loss of *SFRPs* expression leads to aberrant activation of Wnt pathway, which is strongly associated with colorectal carcinogenesis, and is frequently caused by *SFRP2* promoter methylation [32]. Our group recently reported that HCT116 cell line did not express *SFRP2*, which treatment with 5-Aza-2′-deoxycitydine (AZA) recovered *SFRP2* expression [15]. In our study, we found that the *SFRP2* promoter is fully methylated (95% methylation average of the promoter) in the same cell line, suggesting that increased promoter methylation of *SFRP2* could lead to loss/decreased expression of *SFRP2* in CRC patients, which could promote aberrant activation of Wnt signaling pathway.

However, as suggested by our study, higher levels of vitamin D seem to be important in decreasing promotor methylation of *SFRP2*—and increasing *SFRP2* expression as consequence—which could act as Wnt antagonists in CRC patients. However, it is necessary more studies to determine the role of vitamin D in *SFRP2* methylation. Our results suggested that vitamin D could have a possible epigenetic effect, particularly on DNA methylation of *SFRP2* promoter. However, the methylation status may be depending on tissue type, cell type, time of exposure, type of vitamin D active metabolite, and transcriptional analysis of *VDR* and related genes.

According to our results, two studies also found a strong relationship between *SFRP2* and insulin sensitivity. These studies demonstrated that adipose tissue expressions of *SFRP2* and serum *SFRP2* were associated with insulin and HOMA-IR. Thus, *SFRP2* was related to insulin sensitivity and insulin resistance [33, 34]. In our study, *SFRP2* promoter methylation in tumor area was positively correlated with insulin and HOMA-IR in tumor tissue from > 30th CRC patients, but also *SFRP2* methylation in tumor-free area was correlated with HOMA-IR. Our data suggest that *SFRP2* methylation (and loss expression of *SFRP2*) may be a potential candidate in the insulin sensitivity and colorectal cancer axis. The increased insulin sensitivity in tumoral area could promote tumor growth in an independent Wnt signaling pathway (under dependent-insulin pathway), since *SFRP2* is considered an adipokines that promotes insulin sensitivity and insulin resistance [34].

Our results also showed that *SFRP2* methylation was associated with *TIAM1* promoter methylation in CRC tumor area from the > 30th CRC patients. *TIAM1* is considered one of the Wnt responsive genes since that activation/stimulation of Wnt signaling promotes the formation of β-catenin/*TIAM1* complex. This complex is able to activate and enhance Wnt target genes transcription [35]. Recent studies showed that *TIAM1* gene was overexpressed in human colonic adenomas, acting as an oncogene [36], which abnormal promoter methylation of *TIAM1* promoter was tightly associated with aberrant expression in CRC [37]. The further restoration of hypomethylation of *TIAM1* suppresses its expression, cell proliferation, and migration in CRC [37]. The positive association between *SFRP2* and *TIAM1* methylation could suggest similar function. Both *TIAM1* and *SFRP2* hypermethylation leads to decrease expression of both genes and further increase cell proliferation and tumor growth. Thus, this association could display *TIAM1* and *SFRP2* as two candidates to be potential tumor suppressor genes.

*SFRP2* also plays an important role in visceral adipose tissue, more than subcutaneous adipose tissue. A study showed that *SFRP2* did not participate directly in adipogenesis and preadipocyte proliferation, such as *PPARγ* and *C/EBPα*, but treatment with recombinant *SFRP2* was related with adipose tissue through enhanced vascular endothelial growth factor (*VEGF)* expression, which is related with angiogenesis in adipose tissue [33]. *SFRP2* plays a crucial role in contributing to adipocyte function and adipose tissue homeostasis [38]. In our study, in the < 30th CRC patients, promoter methylation *of SFRP2* was negatively associated with methylation status of *C/EBPα* genes in adipose tissue. Indeed, our group previously reported that *C/EBPα* expression was linked to CRC [39]. The association between *SFRP2* and *C/EBPα* could suggest a possible role of *SFRP2* in adipose tissue to CRC carcinogenesis.

Although the exact mechanism by which vitamin D induce changes on DNA methylation is not described yet, its potential to determine methylation marks is a theme to be investigated. A better understanding how vitamin D modulates DNA methylation may help explain the influence of vitamin D on some disease risk. Indeed, low circulating vitamin D is very common in populations, and also low vitamin D levels are associated with many diseases, including CRC. In our data, HCT116 cell treatment with vitamin D did not seem to have any effect in methylation promoter of *SFRP2*, although we found that promoter of *SFRP2* was fully methylated. However, in CRC tumor tissue, the methylation of *SFRP2* was moderately methylated, and higher circulating of vitamin D seems to have close relationship with low methylation rate. Thus, could suggest that vitamin D may have a preventive role. Our results also point towards a possible interaction between vitamin D, *SFRP2* methylation, and neoadjuvant treatments. Only in CRC patients above the 30th percentile of vitamin D, neoadjuvant treatment in a model able to explain up to 24% of the variability, suggesting that higher circulating vitamin D could have an important role in neoadjuvant therapy in CRC patients. Vitamin D may improve the efficacy of neoadjuvant treatment in patients who ensure high circulating vitamin D levels.

## Conclusion

In conclusion, our results showed that higher circulating vitamin D are associated with low *SFRP2* promoter methylation. These results suggested that vitamin D could have an epigenetic effect on DNA methylation. The results of this study open a new avenue to perform further studies in order to elucidate the mechanistic effect that would add novel therapeutic targets for CRC. We need to further explore the effect of vitamin D on *SFRP2* methylation in different normal colon and tumor cell types and tissue type, taking different strategies, such as exposure time, a wide range of doses or consider different type of vitamin D and their analogs or the transcriptional status of VDR and related genes. Finally, we could point to a significant association between neoadjuvant treatment, *SFRP2* methylation, and vitamin D, which could contribute to an improvement response to neoadjuvant treatment.

## Materials and methods

### Study population

In this study, the main analysis was performed in a total of 67 CRC patients, but we included 53 non-CRC participants for partial comparative analysis. All participants were recruited at the “Virgen de la Victoria” University Hospital of Malaga (Málaga, Spain), from 2011 to 2014. The CRC patients were subdivided into two groups based in their 30th percentile of vitamin D, of which 20 of them were below the 30th percentile of vitamin D, and 47 were above the 30th percentile of vitamin D. We selected the 30th percentile of vitamin D, to determine the association of optimal vitamin D and DNA methylation of *SFRP2*, which the > 30th group was according to the optimal 25-OH vitamin D concentrations for colorectal cancer risk reduction, according to a previous study [26]. CRC patients—which were diagnosed by colonoscopy, followed by biopsy, and biopsies samples were classified based on histological diagnoses by pathologists—underwent surgery with curative intention, by hemicolectomy, lower anterior resection with ileostomy (caused by a carcinoma of the CRC), followed by a total mesocolorectal excision. CRC patients were treated according to local protocols. The neoadjuvant treatments were chemoradiation treatments with pelvic radiotherapy 50 Gy (2 Gy/fraction) and concomitant administration of fluoropyrimidine-based chemotherapy, followed by total mesorectal excision in 6–8 weeks. Tumor samples were fixed using formalin-fixed paraffin-embedded (FFPE). We used 10 sections of 14 μm from tumor area and an adjacent tumor-free area from all CRC patients. Epiploic VAT from CRC patients and non-CRC participants was obtained during surgery, washed in physiological saline solution, and immediately frozen in liquid nitrogen. Biopsy samples were maintained at − 80 °C until analysis. PBMCs were obtained from whole blood and were separated by centrifugation for 15 min at 4000 rpm and immediately frozen at − 80 °C until DNA extraction. The exclusion criteria were patients with acute or chronic inflammatory diseases, cardiovascular diseases, hereditary non-polyposis colorectal cancer or familial adenomatous polyposis, type 2 diabetes, or renal and infectious diseases. We also excluded who had taken treatment that alter the lipid or glucose metabolism or who consumed > 20 g of ethanol/day. Written informed consent was obtained from all patients and subjects. The study was reviewed and approved by the Ethics Committees of Virgen de la Victoria University Hospital (Málaga, Spain) (Registration number 0311/PI7).

### Biochemical determination

Fasting blood samples from all subjects were obtained before surgery. Serum samples were separated by centrifugation for 15 min at 4000 rpm. Serum glucose, triglycerides, total cholesterol, and high-density lipoprotein cholesterol (HDL-c) and alkaline phosphatase were measured using Dimension Autoanalyzer (Dade Behring Inc.). Calculated value of low density lipoprotein cholesterol (LDL-c) was obtained using the Friedewald equation [40]. Insulin levels were quantified by radioimmunoassay methods using BioSource International Inc. (Camarillo, CA, USA). Corrected calcium was obtained under the equation: fasting calcium (mg/dl) + 0.8 × (4-fasting albumin (g/dl)). The homeostasis model assessment of insulin resistance (HOMA-IR) was obtained using the following equation: HOMA-IR = fasting insulin (μIU/ml) × fasting glucose (mmol/l)/22.5 [41]. Serum 25-OH vitamin D was quantified by ELISA kit (Immundiagnostik, Bensheim, Germany).

### DNA extraction, bisulfite reaction, and pyrosequencing

DNA from PBMCs was isolated using DNA Qiamp Blood Mini Kit (Qiagen GmbH, Hilden, Germany) following the manufacturer’s instructions. DNA from VAT was obtained using Qiamp DNA Tissue Kit (Qiagen GmbH, Hilden, Germany) according to the manufacturer’s guidelines (Qiagen GmbH, Hilden, Germany). Paraffin samples from tumor area and tumor-free area were isolated using Qiamp DNA FFPE Tissue Kit under the instructions of the manufacturer (Qiagen GmbH, Hilden, Germany), with a xylene wash, to remove the paraffin.

Two micrograms of DNA genomic isolated was used for bisulfite reaction, using EpiTect Fast Bisulfite Kit (Qiagen GmbH, Hilden, Germany), following the manufacturer instructions. The bisulfited DNA was used to determine DNA methylation using the PyroMark Q96 ID pyrosequencing System (Qiagen GmbH, Hilden, Germany). The reference numbers for premade PyroMark CpG assay for *VDR* and *NFκB* are PM00110908 and PM00051443, respectively. The primer sequences and data about CpG sites for *SFRP2*, *TIAM1*, *ZNF397OS*, *ZNF543*, *C/EBPα*, *PPAR*γ, *PGC1α*, and *TNFα* genes are detailed in the Table S2 (for a graphical representation of the promoter of the studied genes, please see Figure S1).

PCR reaction was made using 0.2 mol/l of the concentration of primers in a total volume of 25 μl. PCR products were purified using pyrosequencing Vacuum Prep-Tool (Qiagen GmbH, Hilden, Germany), and DNA sequencing was carried out using the Pyromark TMQ96 ID Pyrosequencing system (Qiagen GmbH, Hilden, Germany). The methylation average was presented as the percentage of methylated cytosine over the sum of methylated and unmethylated cytosines. Interassay precision (%CV) was 2.5% and intraassay (%CV) was 1.0%. Non-CpG cytosine residues were used as built-in controls to verify bisulfite conversion. We also used unmethylated and methylated DNA as controls in our assay (New England Biolabs, UK).

### Culture cells

Human colorectal carcinoma cell lines (HCT116, Homo Sapiens colon colorectal carcinoma, ATCC, Manassas, VA, USA), were used to study the promoter methylation of *SFRP2* under treatment with vitamin D. Cells were cultured in Dulbecco’s modified Eagle’s medium containing 10% fetal bovine serum, penicillin, and streptomycin at 37 °C and 5% CO2. Cell lines were treated with the vitamin D (Sigma Aldrich, Madrid, Spain) at 10 nM and 100 nM for 2, 6, 12, 24, 48, and 72 h.

### Statistical analysis

The results are shown as mean ± standard deviation (SD) for continuous variables and as number/percentages for non-continuous variables. We used Welch two sample tests to determine difference between methylation variables due the sample size and non-normal distribution. In addition, Student *t*-test or Wilcoxon test was applied according to normality to anthropometric and biochemical variables. A Pearson’s correlation analysis was performed to evaluate whether the methylation of the different genes was correlated between them and with anthropometric and biochemical parameters. Linear regression analysis was performed to determine whether *SFRP2* methylation is explained by other variables, under the 30th percentile vitamin D. Analyses were performed using the R v.3.5.1 software, and significance was set at *p* < 0.05.

## Supplementary information

**Additional file 1: Figure S1.** Promoter overview of the selected genes, generated by UCSC genome Browser (https://genome.ucsc.edu). The sequence analyzed is highlighted in light blue of SFRP-2 (a), TIAM1 (b), ZNF397OS (c), ZNF543 (d), C/EBPα (e), PPAR-γ (f), PGC-1α (g), NF-κB (h), TNF-α (i) and VDR (j).

**Additional file 2: Figure S2.** Representation of methylation status of selected genes under the 30th percentile of vitamin D. a) Methylation status of genes in tumoral tissue. b) Methylation status in visceral adipose tissue.

**Additional file 3: Figure S3.** The effect of 25-OH vitamin D in the SFRP-2 promoter methylation in HCT116 cell line. SFRP-2 promoter methylation in HCT116 after treatment with 25-OH-Vitamin D during 2, 6, 12, 24, 48 and 72 hours under 10 and 100 nM. The results are given as the methylation average mean and standard deviation. Abbreviations: SFRP2: Secreted frizzled-related protein type 2; HCT116: Homo sapiens colon colorectal carcinoma.

**Additional file 4: Table S1.**

**Additional file 5: Table S2.** Data of CpG analyzed and primer sequences of selected promoter

**Additional file 6: Table S3**. General data, clinical and biochemical variables of control and CRC patients.

## Data Availability

Data will not be shared. An explicit statement was not included in the informed consent to allow data sharing. Data were not collected anonymously. Research data are stored with patients’ name, contact information, or another identifier.

## Abbreviations

CRC: Colorectal cancer
Wnt: Wingless
SFRP: Secreted frizzled-related protein
APC: Adenomatous polyposis coli
LINE1: Genomic long interspersed nuclear element-1
DKK1: Dickkopf1
PBMCs: Peripheral blood mononuclear cells
VAT: Visceral adipose tissue
HCT116: Human colorectal carcinoma cell lines 116
TIAM1: T cell lymphoma invasion and metastasis 1
ZNF397OS: Zinc finger protein type 397OS
C/EBPα: CCAAT/enhancer-binding protein alpha
PPARγ: Peroxisome proliferator activated receptor gamma
PGC1α: Peroxisome proliferator activated receptor gamma coactivator 1 alpha
NFκB: Nuclear factor kappa B
TNFα: Tumor necrosis factor alpha
VDR: Vitamin D receptor
PTEN: Phosphatase and tensin homolog
CDH1: Cadherin 1
AZA: 5-AZA-20-deoxycitydine

## References

[1] B. W. Stewart and C. P. Wild, “World cancer report 2014,” World Heal. Organ., 2014, doi: 9283204298.

[2] K. Bardhan and K. Liu, “Epigenetics and colorectal cancer pathogenesis,” Cancers (Basel)., 2013, doi: 10.3390/cancers5020676. 10.3390/cancers5020676 PMC3730326 24216997

[3] I. Mármol, C. Sánchez-de-Diego, A. P. Dieste, E. Cerrada, and M. J. R. Yoldi, “Colorectal carcinoma: a general overview and future perspectives in colorectal cancer,” International Journal of Molecular Sciences. 2017, doi: 10.3390/ijms18010197. 10.3390/ijms18010197 PMC5297828 28106826

[4] H. van Andel, K. A. Kocemba, M. Spaargaren, and S. T. Pals, “Aberrant Wnt signaling in multiple myeloma: molecular mechanisms and targeting options,” Leukemia. 2019, doi: 10.1038/s41375-019-0404-1. 10.1038/s41375-019-0404-1 PMC6756057 30770859

[5] S. G. Pai et al., “Wnt/beta-catenin pathway: modulating anticancer immune response,” Journal of Hematology and Oncology. 2017, doi: 10.1186/s13045-017-0471-6. 10.1186/s13045-017-0471-6 PMC5420131 28476164

[6] A. Rattner *et al.*, “A family of secreted proteins contains homology to the cysteine-rich ligand-binding domain of frizzled receptors,” Proc. Natl. Acad. Sci. U. S. A., 1997, doi: 10.1073/pnas.94.7.2859. 10.1073/pnas.94.7.2859 PMC20287 9096311

[7] Lin K, Wang S, Julius MA, Kitajewski J, Moos M, Luyten FP. The cysteine-rich frizzled domain of Frzb-1 is required and sufficient for modulation of Wnt signaling. Proc. Natl. Acad. Sci. U. S. A. 1997. 10.1073/pnas.94.21.11196. 10.1073/pnas.94.21.11196 PMC23413 9326585

[8] Jithesh PV, et al. The epigenetic landscape of oral squamous cell carcinoma. *Br. J. Cancer*. 2013. 10.1038/bjc.2012.568. 10.1038/bjc.2012.568 PMC3566828 23287992

[9] H. Takagi *et al.*, “Frequent epigenetic inactivation of SFRP genes in hepatocellular carcinoma,” J. Gastroenterol., 2008, doi: 10.1007/s00535-008-2170-0. 10.1007/s00535-008-2170-0 18592156

[10] Cheng YY, et al. Frequent epigenetic inactivation of secreted frizzled-related protein 2 (SFRP2) by promoter methylation in human gastric cancer. *Br. J. Cancer*. 2007. 10.1038/sj.bjc.6603968. 10.1038/sj.bjc.6603968 PMC2360406 17848950

[11] Q. Xiao, Y. Yang, X. Zhang, and Q. An, “Enhanced Wnt signaling by methylation-mediated loss of SFRP2 promotes osteosarcoma cell invasion,” Tumor Biol., 2016, doi: 10.1007/s13277-015-4466-z. 10.1007/s13277-015-4466-z 26628297

[12] J. Veeck *et al.*, “Promoter hypermethylation of the SFRP2 gene is a high-frequent alteration and tumor-specific epigenetic marker in human breast cancer,” Mol. Cancer, 2008, doi: 10.1186/1476-4598-7-83. 10.1186/1476-4598-7-83 PMC2613402 18990230

[13] Q. Yang, T. Huang, G. Ye, B. Wang, and X. Zhang, “Methylation of SFRP2 gene as a promising noninvasive biomarker using feces in colorectal cancer diagnosis: a systematic meta-analysis,” Sci. Rep., 2016, doi: 10.1038/srep33339. 10.1038/srep33339 PMC5034263 27659069

[14] H. Zhang, Y. Q. Zhu, Y. Q. Wu, P. Zhang, and J. Qi, “Detection of promoter hypermethylation of Wnt antagonist genes in fecal samples for diagnosis of early colorectal cancer,” World J. Gastroenterol., 2014, doi: 10.3748/wjg.v20.i20.6329. 10.3748/wjg.v20.i20.6329 PMC4033472 24876755

[15] A. Cabrera-Mulero *et al.*, “Novel SFRP2 DNA methylation profile following neoadjuvant therapy in colorectal cancer patients with different grades of BMI.,” *J. Clin. Med.*, vol. 8, no. 7, 2019, doi: 10.3390/jcm8071041. 10.3390/jcm8071041 PMC6678889 31319558

[16] H. G. Pálmer *et al.*, “Vitamin D3 promotes the differentiation of colon carcinoma cells by the induction of E-cadherin and the inhibition of β-catenin signaling,” J. Cell Biol., 2001, doi: 10.1083/jcb.200102028. 10.1083/jcb.200102028 PMC2150773 11470825

[17] O. Aguilera *et al.*, “The Wnt antagonist DICKKOPF-1 gene is induced by 1α,25-dihydroxyvitamin D3 associated to the differentiation of human colon cancer cells,” Carcinogenesis, 2007, doi: 10.1093/carcin/bgm094. 10.1093/carcin/bgm094 17449905

[18] J. Wang *et al.*, “20-Hydroxyvitamin D 3 inhibits proliferation of cancer cells with high efficacy while being non-toxic,” Anticancer Res., 2012. PMC3312810 22399586

[19] I. S. Fetahu, J. Höbaus, and E. Kállay, “Vitamin D and the epigenome,” *Frontiers in Physiology*. 2014, doi: 10.3389/fphys.2014.00164. 10.3389/fphys.2014.00164 PMC4010791 24808866

[20] H. S. Tapp *et al.*, “Nutritional factors and gender influence age-related DNA methylation in the human rectal mucosa,” Aging Cell, 2013, doi: 10.1111/acel.12030. 10.1111/acel.12030 PMC3572581 23157586

[21] Rawson JB, et al. Vitamin D intake is negatively associated with promoter methylation of the Wnt antagonist gene DKK1 in a large group of colorectal cancer patients. *Nutr. Cancer*. 2012. 10.1080/01635581.2012.711418. 10.1080/01635581.2012.711418 PMC4323165 22966878

[22] Y. Ma, D. L. Trump, and C. S. Johnson, “Vitamin D in combination cancer treatment,” *Journal of Cancer*. 2010, doi: 10.7150/jca.1.101. 10.7150/jca.1.101 PMC2938072 20842231

[23] K. Ng *et al.*, “Effect of high-dose vs standard-dose vitamin D3 supplementation on progression-free survival among patients with advanced or metastatic colorectal cancer: the SUNSHINE randomized clinical trial,” JAMA, 2019, doi: 10.1001/jama.2019.2402. 10.1001/jama.2019.2402 PMC6459117 30964527

[24] A. B. Crujeiras *et al.*, “Identification of an episignature of human colorectal cancer associated with obesity by genome-wide DNA methylation analysis,” Int. J. Obes., 2018, doi: 10.1038/s41366-018-0065-6. 10.1038/s41366-018-0065-6 29717273

[25] S. M. Jeon and E. A. Shin, “Exploring vitamin D metabolism and function in cancer,” *Experimental and Molecular Medicine*. 2018, doi: 10.1038/s12276-018-0038-9. 10.1038/s12276-018-0038-9 PMC5938036 29657326

[26] M. L. McCullough *et al.*, “Circulating vitamin D and colorectal cancer risk: an international pooling project of 17 cohorts,” J. Natl. Cancer Inst., 2019, doi: 10.1093/jnci/djy087. 10.1093/jnci/djy087 PMC6376911 29912394

[27] A. Saramäki, S. Diermeler, R. Kellner, H. Laitinen, S. Väisänen, and C. Cariberg, “Cyclical chromatin looping and transcription factor association on the regulatory regions of the p21 (CDKN1A) gene in response to 1α,25-dihydroxyvitamin D3,” J. Biol. Chem*.*, 2009, doi: 10.1074/jbc.M808090200. 10.1074/jbc.M808090200 PMC2658101 19122196

[28] B. Stefanska, H. Karlic, F. Varga, K. Fabianowska-Majewska, and A. G. Haslberger, “Epigenetic mechanisms in anti-cancer actions of bioactive food components - the implications in cancer prevention,” *British Journal of Pharmacology*. 2012, doi: 10.1111/j.1476-5381.2012.02002.x. 10.1111/j.1476-5381.2012.02002.x PMC3481038 22536923

[29] I. S. Fetahu, J. Hobaus, and E. Kallay, “Vitamin D and the epigenome,” *Frontiers in Physiology*. 2014, doi: 10.3389/fphys.2014.00164. 10.3389/fphys.2014.00164 PMC4010791 24808866

[30] J. B. Rawson *et al.*, “Promoter methylation of Wnt antagonists DKK1 and SFRP1 is associated with opposing tumor subtypes in two large populations of colorectal cancer patients,” Carcinogenesis, 2011, doi: 10.1093/carcin/bgr020. 10.1093/carcin/bgr020 PMC3140140 21304055

[31] P. Bovolenta, P. Esteve, J. M. Ruiz, E. Cisneros, and J. Lopez-Rios, “Beyond Wnt inhibition: new functions of secreted Frizzled-related proteins in development and disease,” *Journal of Cell Science*. 2008, doi: 10.1242/jcs.026096. 10.1242/jcs.026096 18322270

[32] H. Suzuki, M. Toyota, M. Nojima, M. Mori, and K. Imai, “SFRP, a family of new colorectal tumor suppressor candidate genes,” *Nippon rinsho. Japanese journal of clinical medicine*. 2005. 15828241

[33] R. K. Crowley *et al.*, “SFRP2 is associated with increased adiposity and VEGF expression,” PLoS One, 2016, doi: 10.1371/journal.pone.0163777. 10.1371/journal.pone.0163777 PMC5042473 27685706

[34] A. Ehrlund *et al.*, “Characterization of the Wnt inhibitors secreted frizzled-related proteins (SFRPs) in human adipose tissue,” J. Clin. Endocrinol. Metab., 2013, doi: 10.1210/jc.2012-3416. 10.1210/jc.2012-3416 23393180

[35] P. Buongiorno, V. V. Pethe, G. S. Charames, S. Esufali, and B. Bapat, “Rac1 GTPase and the Rac1 exchange factor Tiam1 associate with Wnt-responsive promoters to enhance beta-catenin/TCF-dependent transcription in colorectal cancer cells,” Mol. Cancer, 2008, doi: 10.1186/1476-4598-7-73. 10.1186/1476-4598-7-73 PMC2565678 18826597

[36] A. Malliri *et al.*, “The Rac activator Tiam1 is a Wnt-responsive gene that modifies intestinal tumor development,” J. Biol. Chem., 2006, doi: 10.1074/jbc.M507582200. 10.1074/jbc.M507582200 16249175

[37] R. Du *et al.*, “Reversing hypomethylation of TIAM1 promoter inhibits TIAM1 gene expression and cell proliferation and migration in colorectal cancer,” Int. J. Clin. Exp. Pathol., 2016.

[38] J. Park, M. Kim, K. Sun, Y. A. An, X. Gu, and P. E. Scherer, “VEGF-A - expressing adipose tissue shows rapid beiging and enhanced survival after transplantation and confers IL-4-independent metabolic improvements,” Diabetes, 2017, doi: 10.2337/db16-1081. 10.2337/db16-1081 PMC5440018 28254844

[39] H. Boughanem *et al.*, “The expression/methylation profile of adipogenic and inflammatory transcription factors in adipose tissue are linked to obesity-related colorectal cancer,” *Cancers (Basel).*, 2019, doi: 10.3390/cancers11111629. 10.3390/cancers11111629 PMC6893417 31652933

[40] W. T. Friedewald, R. I. Levy, and D. S. Fredrickson, “Estimation of the concentration of low-density lipoproteisn cholesterol in plasma, without use of the preparative ultracentrifuge.,” Clin. Chem., 1972, doi: 10.1177/107424840501000106. 4337382

[41] D. R. Matthews, J. P. Hosker, A. S. Rudenski, B. A. Naylor, D. F. Treacher, and R. C. Turner, “Homeostasis model assessment: insulin resistance and β-cell function from fasting plasma glucose and insulin concentrations in man,” Diabetologia, 1985, doi: 10.1007/BF00280883. 10.1007/BF00280883 3899825

