# Supplementary material for: Association between variation of circulating 25-OH vitamin D and methylation of secreted frizzled-related protein 2 in colorectal cancer
Source: Clin Epigenetics. 2020 Jun 9;12:83. doi: 10.1186/s13148-020-00875-9 (PMC7285750; doi:10.1186/s13148-020-00875-9)
Supplement: Supplementary file 1 — Additional file 1: Figure S1. Promoter overview of the selected genes, generated by UCSC genome Browser (https://genome.ucsc.edu). The sequence analyzed is highlighted in light blue of SFRP-2 (a), TIAM1 (b), ZNF397OS (c), ZNF543 (d), C/EBPα (e), PPAR-γ (f), PGC-1α (g), NF-κB (h), TNF-α (i) and VDR (j). [file 13148_2020_875_MOESM1_ESM.pdf]

Figure S1. Promoter overview of the selected genes, generated by UCSC genome Browser (https://genome.ucsc.edu). The sequence analyzed is highlighted in light blue of SFRP-2 (a), TIAM1 (b), ZNF397OS (c), ZNF543 (d), C/EBPα (e), PPAR-γ (f), PGC-1α (g), NF-κB (h), TNF-α (i) and VDR (j).

a) SFRP-2

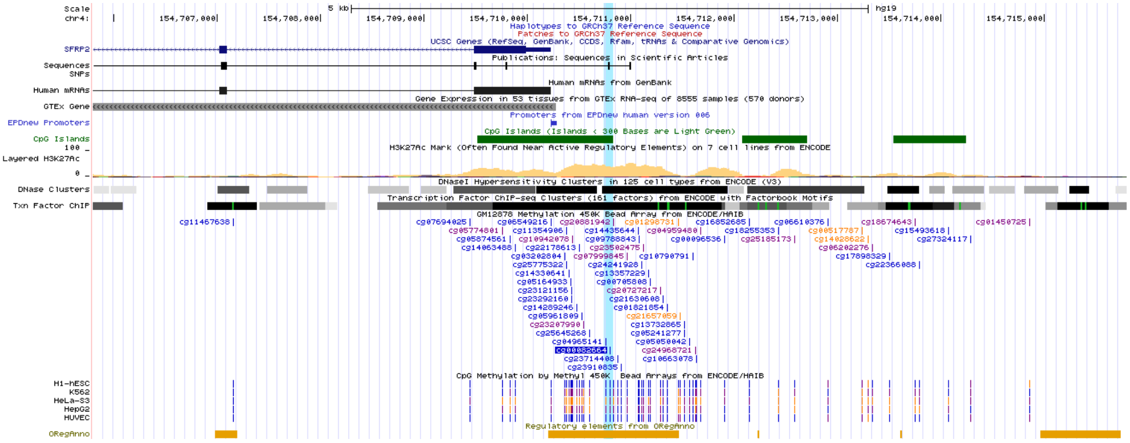

b) TIAM1

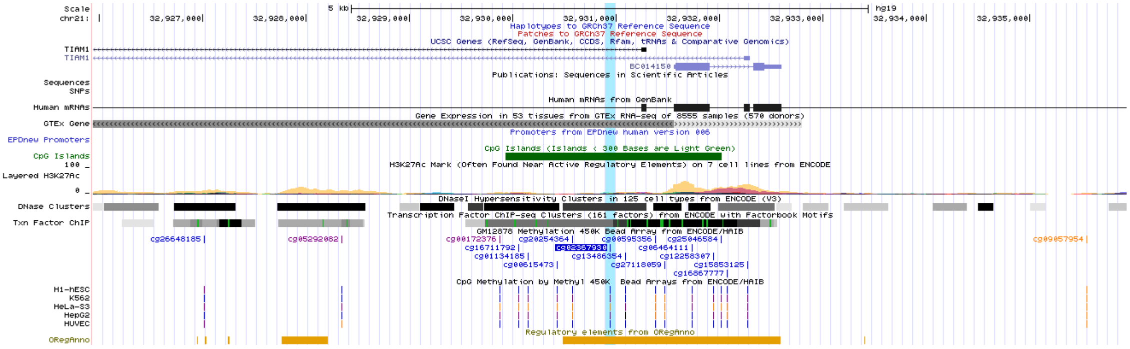

c) ZNF397OS

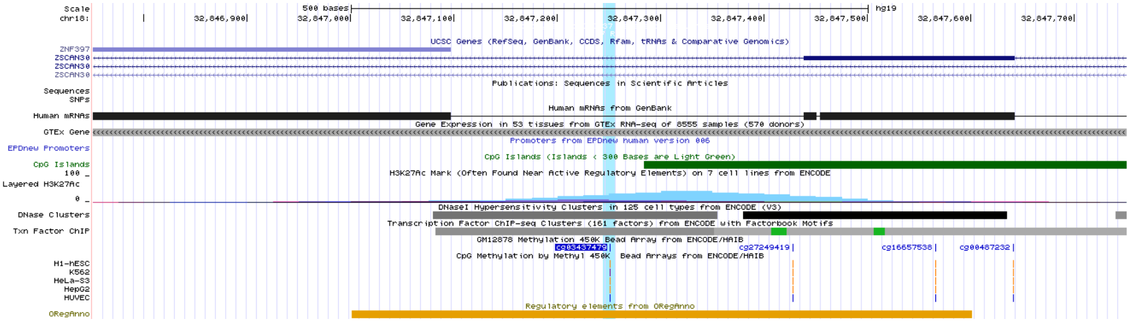

d) ZNF543

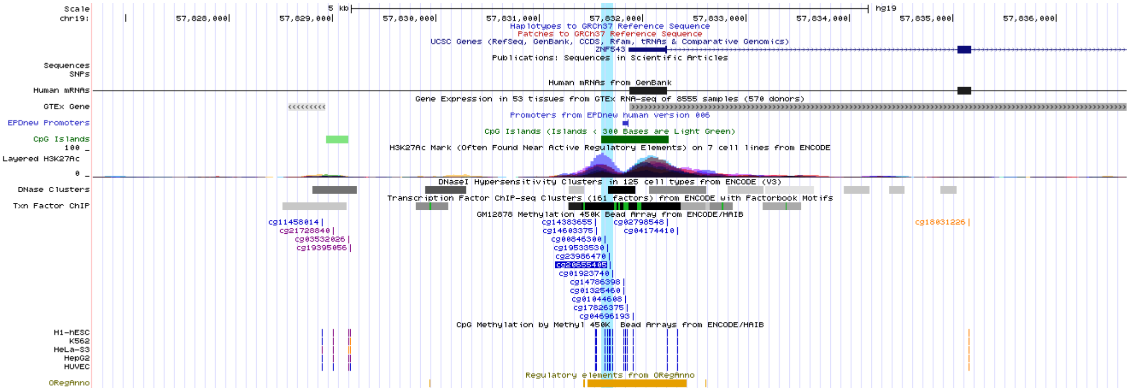

e) C/EBPα

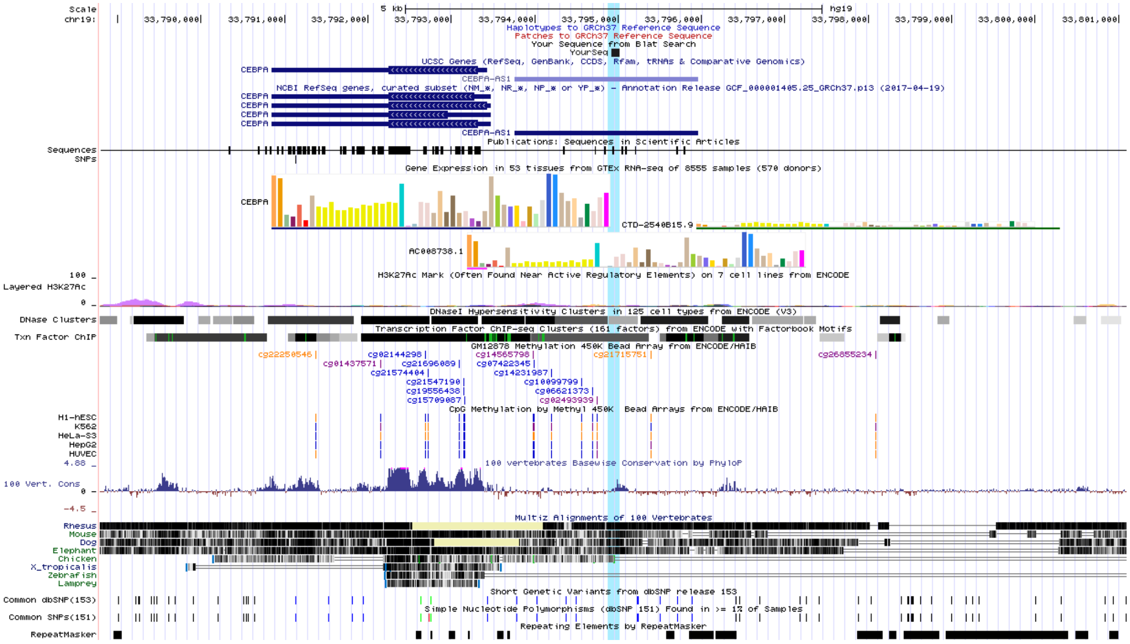

f) PPAR-γ

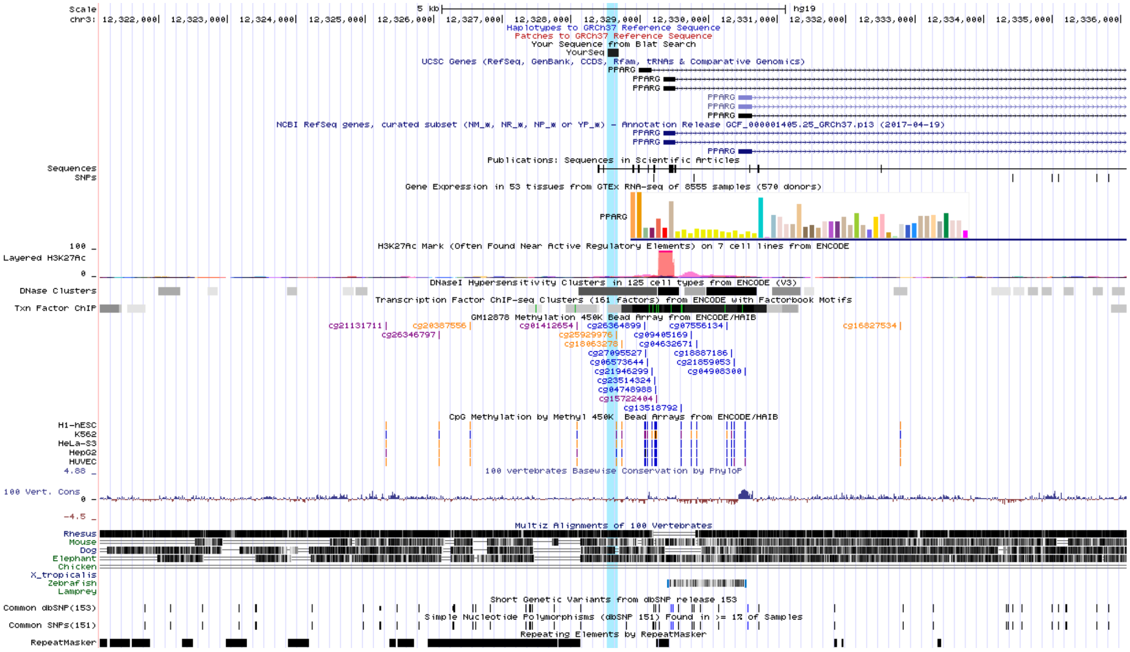

g) PGC-1α

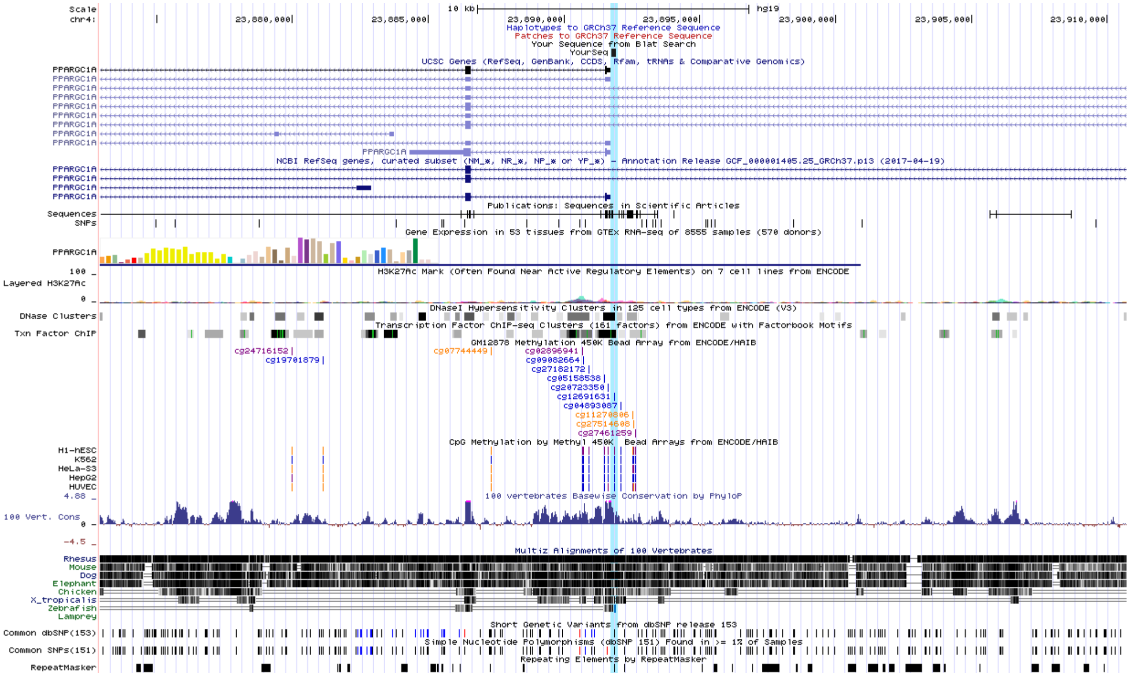

h) NF-κB

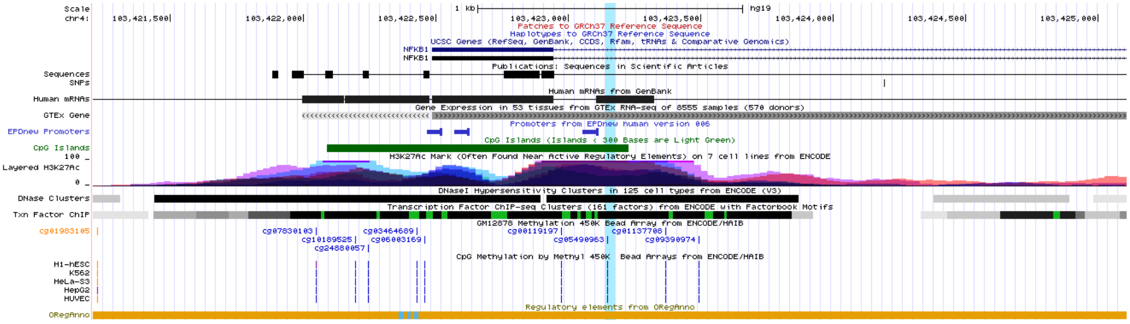

Scale
chr4:
hg19
NFKB1
Sequences
SNPs
Human mRNAs
GTEx Gene
EPDnew Promoters
CpG Islands
Layered H3K27Ac
DNase Clusters
Txn Factor ChIP
H1-hESC
K562
HeLa-S3
HepG2
HUVEC
ORegAnno

i) TNF-α

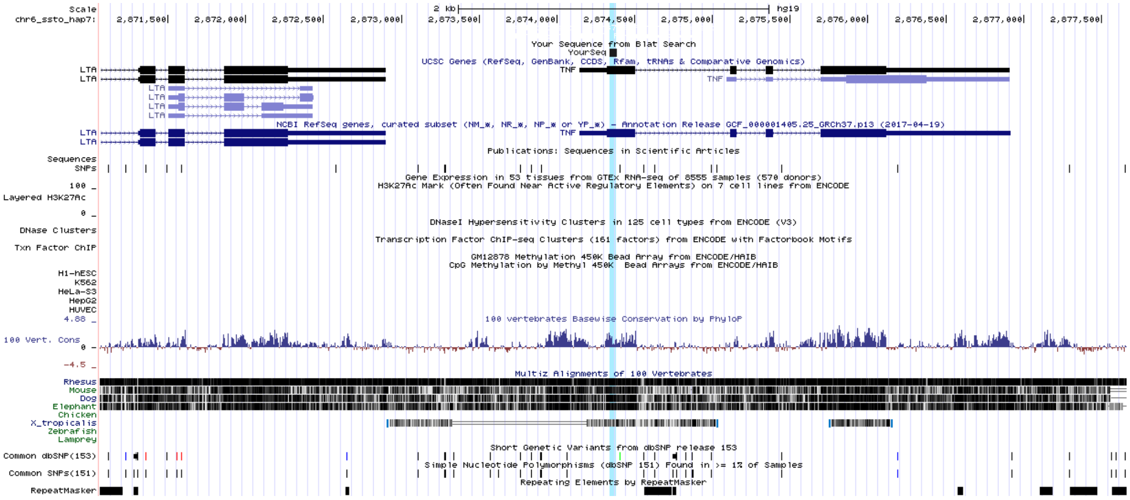

Scale
chr6_ssto_hap7:
hg19
LTA
TNF
Sequences
SNPs
Layered H3K27Ac
DNase Clusters
Txn Factor ChIP
H1-hESC
K562
HeLa-S3
HepG2
HUVEC
100 Vert. Cons
Rhesus
Mouse
Dog
Elephant
Chicken
X_tropicalis
Zebrafish
Lamprey
Common dbSNP(153)
Common SNPs(151)
RepeatMasker

j) VDR

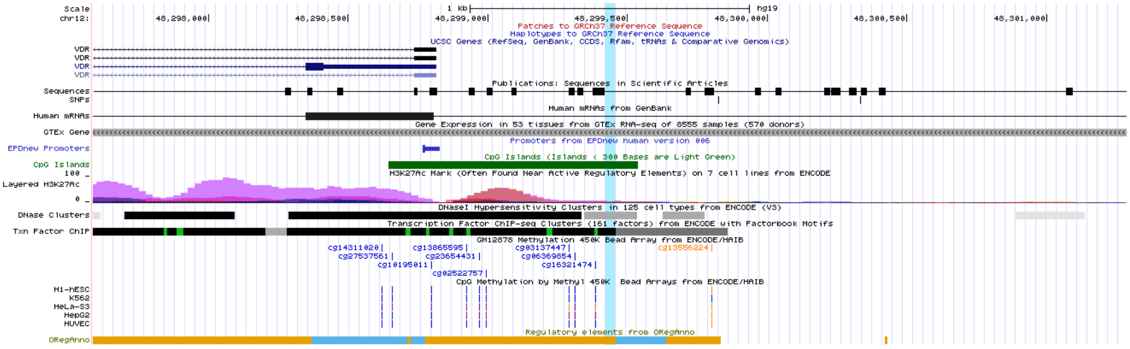

Scale
chr12:
hg19
VDR
Sequences
SNPs
Human mRNAs
GTEx Gene
EPDnew Promoters
CpG Islands
Layered H3K27Ac
DNase Clusters
Txn Factor ChIP
H1-hESC
K562
HeLa-S3
HepG2
HUVEC
ORegAnno
