# Supplementary material for: Association between variation of circulating 25-OH vitamin D and methylation of secreted frizzled-related protein 2 in colorectal cancer
Source: Clin Epigenetics. 2020 Jun 9;12:83. doi: 10.1186/s13148-020-00875-9 (PMC7285750; doi:10.1186/s13148-020-00875-9)
Supplement: Supplementary file 2 — Additional file 2: Figure S2. Representation of methylation status of selected genes under the 30th percentile of vitamin D. a) Methylation status of genes in tumoral tissue. b) Methylation status in visceral adipose tissue. [file 13148_2020_875_MOESM2_ESM.pdf]

a)

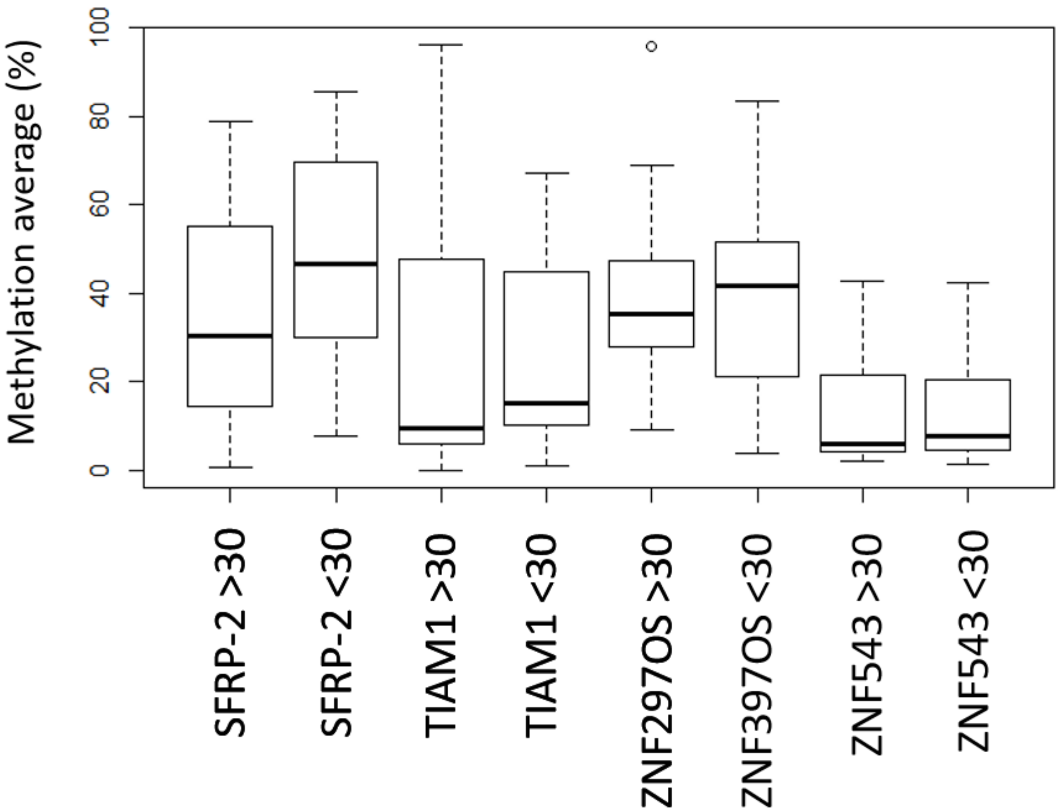
Methylation average (%)
0
20
40
60
80
100
SFRP-2 >30
SFRP-2 <30
TIAM1 >30
TIAM1 <30
ZNF297OS >30
ZNF397OS <30
ZNF543 >30
ZNF543 <30

b)

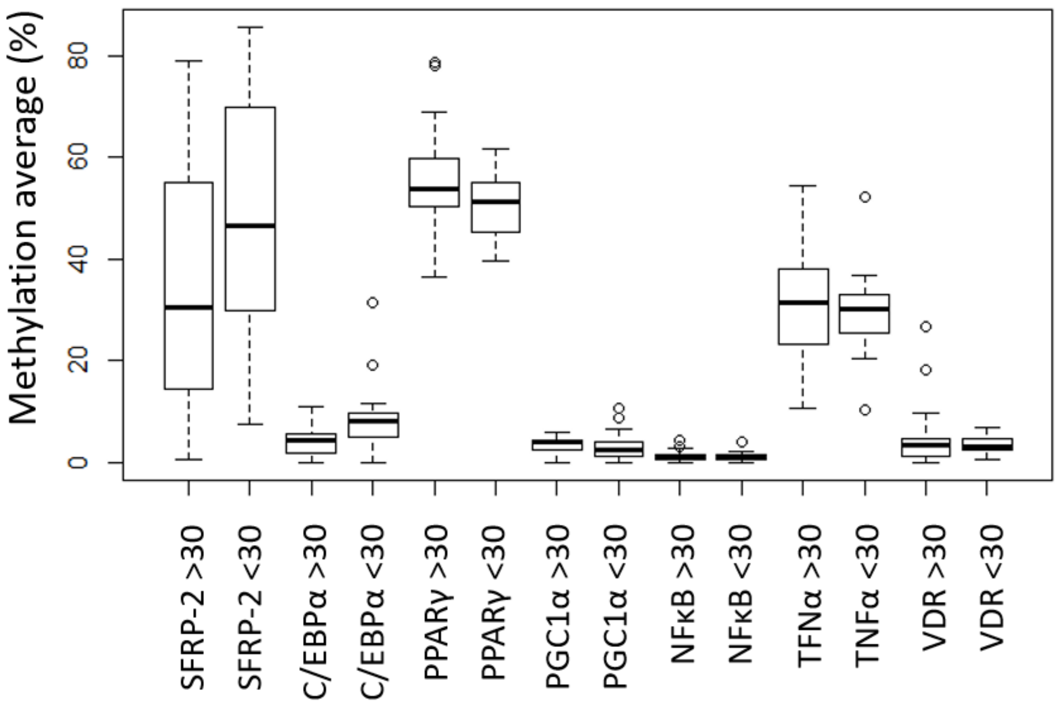

Figure S2. Representation of methylation status of selected genes under the 30th percentile of vitamin D. a) Methylation status of genes in tumoral tissue. b) Methylation status in visceral adipose tissue.
