# Supplementary material for: Association between variation of circulating 25-OH vitamin D and methylation of secreted frizzled-related protein 2 in colorectal cancer
Source: Clin Epigenetics. 2020 Jun 9;12:83. doi: 10.1186/s13148-020-00875-9 (PMC7285750; doi:10.1186/s13148-020-00875-9)
Supplement: Supplementary file 3 — Additional file 3: Figure S3. The effect of 25-OH vitamin D in the SFRP-2 promoter methylation in HCT116 cell line. SFRP-2 promoter methylation in HCT116 after treatment with 25-OH-Vitamin D during 2, 6, 12, 24, 48 and 72 hours under 10 and 100 nM. The results are given as the methylation average mean and standard deviation. Abbreviations: SFRP2: Secreted frizzled-related protein type 2; HCT116: Homo sapiens colon colorectal carcinoma. [file 13148_2020_875_MOESM3_ESM.pdf]

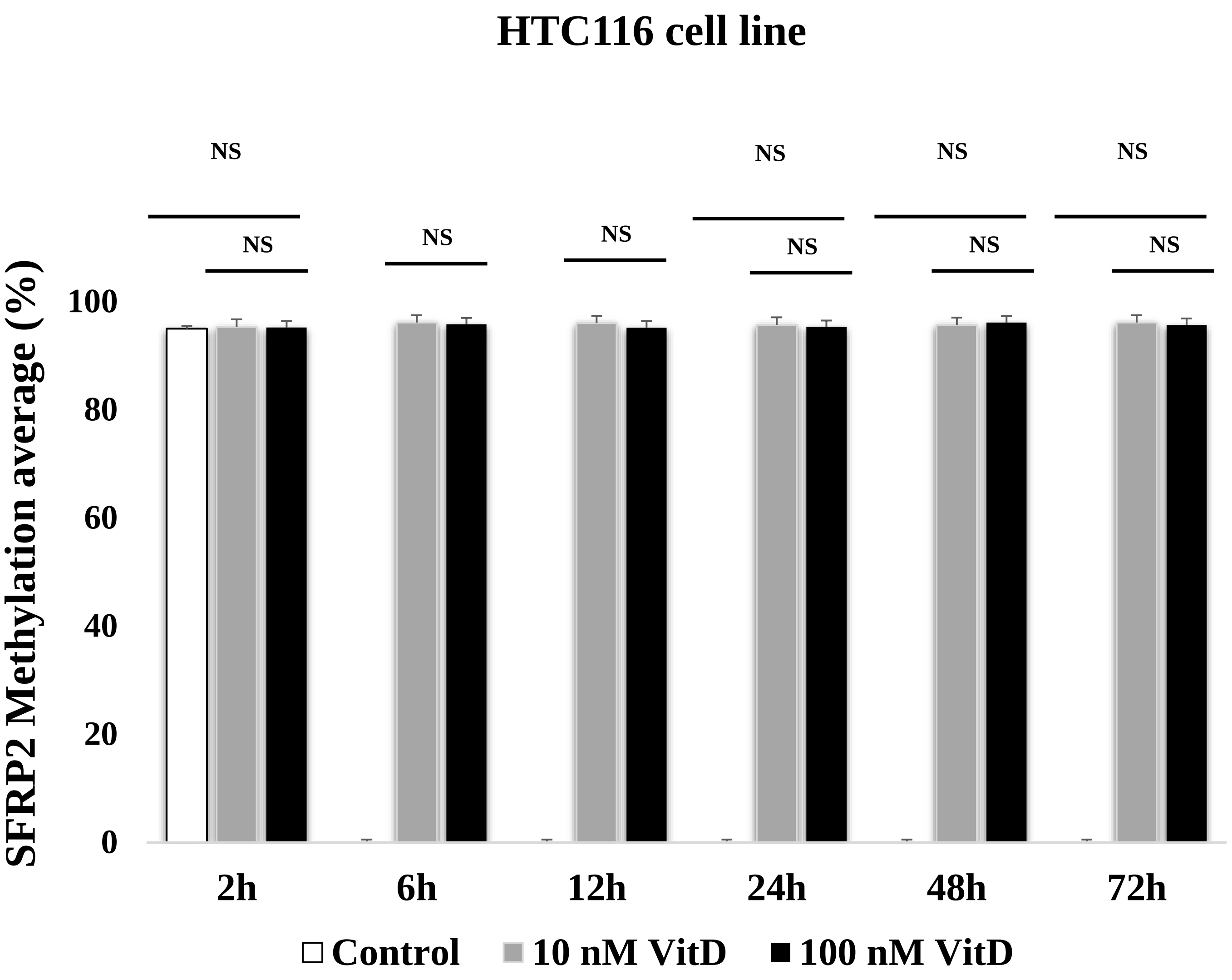

Figure S3. The effect of 25-OH vitamin D in the SFRP-2 promoter methylation in HCT116 cell line.
SFRP-2 promoter methylation in HCT116 after treatment with 25-OH-Vitamin D during 2, 6, 12, 24, 48 and 72 hours under 10 and 100 nM. The results are given as the methylation average mean and standard deviation. **Abbreviations**: SFRP2: Secreted frizzled-related protein type 2; HCT116: Homo sapiens colon colorectal carcinoma.
