# Supplementary material for: Association between variation of circulating 25-OH vitamin D and methylation of secreted frizzled-related protein 2 in colorectal cancer
Source: Clin Epigenetics. 2020 Jun 9;12:83. doi: 10.1186/s13148-020-00875-9 (PMC7285750; doi:10.1186/s13148-020-00875-9)
Supplement: Supplementary file 4 — Additional file 4: Table S1. [file 13148_2020_875_MOESM4_ESM.pdf]

Variables	SFRP-2 PBMCs		SFRP-2 VAT		SFRP-2 tumor area		SFRP-2 tumor area free	
**25-OH-Vitamin D percentiles**	<30th	>30th	<30th	>30th	<30th	>30th	<30th	>30th
Age (years)	0.12	**0.12***	0.37	0.37	0.16	0.15	-0.02	-0.02
Gender	0.38	**0.36***	**-0.52***	-0.52	-0.08	-0.06	-0.16	-0.15
**Anthropometric variables**								
BMI (kg/m^2)	0.24	0.24	-0.18	-0.16	0.08	0.08	0.43	0.43
Waist circumference (cm)	0.69	0.59	0.10	0.1	-0.28	-0.26	0.16	0.15
**Glucose metabolism**								
Glucose (mg/dl)	0.10	0.10	0.07	0.07	-0.02	-0.02	0.03	0.03
Insulin (μUI/ml)	-0.19	-0.19	0.12	0.12	0.27	**0.27***	0.19	0.19
HOMA-IR	-0.11	-0.11	-0.32	-0.32	0.23	**0.23***	0.38	**0.36***
**Lipid metabolism**								
Triglycerides (mg/dl)	-0.11	-0.11	-0.16	-0.15	0.18	0.19	0.13	-0.02
Total cholesterol (mg/dl)	0.27	0.27	-0.12	-0.12	-0.26	-0.25	-0.26	0.13
HDL-c (mg/dl)	0.08	0.08	0.14	0.14	-0.31	**-0.31***	-0.18	-0.16
LDL-c (mg/dl)	0.38	0.36	-0.28	-0.29	-0.20	-0.2	-0.27	-0.27
