# Supplementary material for: Association between variation of circulating 25-OH vitamin D and methylation of secreted frizzled-related protein 2 in colorectal cancer
Source: Clin Epigenetics. 2020 Jun 9;12:83. doi: 10.1186/s13148-020-00875-9 (PMC7285750; doi:10.1186/s13148-020-00875-9)
Supplement: Supplementary file 5 — Additional file 5: Table S2. Data of CpG analyzed and primer sequences of selected promoter [file 13148_2020_875_MOESM5_ESM.pdf]

Table S2: Data of CpG analyzed and primer sequences of selected promoter.

Gene	Position	Primer sequences	Number of analyzed CpG sites
SFRP-2	Chromosome 4<br>154,710,796	F: AGAAGTTTTGGGTTTAGTTTATGAT<br>R: CTCACATCTACCCAATATAAAAACTCACCA<br>S1: TGTTGAAYGGTGGTTGG	7
TIAM1	Chromosome 21<br>32,930,938	F: GAGATGYGGGGAGGAGAGG<br>R: CAAATCAACRCTAACCTCCTACT<br>S1: GTAGGAGGAGGTATTTTT	7
ZNF397OS	Chromosome 18<br>32,847,251	F: GGGGTTYGGTTGTATTTGGG<br>R: ATATCAAACTCCTAAACCTACAAC<br>S1: GGTTAGTTTYGTTATTTATTTTTA	1
ZNF543	Chromosome 19<br>57,831,684	F: AGAAGTTTTGGGTTTAGTTTATGAT<br>R: CTCACATCTACCCAATATAAAAACTCACCA<br>S1: ATTTTTTTAAGTTAGAAAGGATT	8
C/EBP-α	Chromosome 19<br>33,794,959	F: TGGGACTATGTTGAATAGGAACTTG<br>R: GGAGACACTTGAGGGCTCCCAA<br>S1: GTGCCCCCTCCGGGGCTCCTGG	4
PPAR-γ	Chromosome 3<br>12,328,531	F: AAGAGGATCAGGCCCAGAACAG<br>R: CACTGTCTAC<br>S1: CAGCGGCTTTCTGAAC	5
PGC-1α	Chromosome 4<br>23,891,933	F: AGGGGATTTTGGTTATTATATGGCCA<br>R: AGGCTGGGTGAGTGACAGCCCAG<br>S1: TTTAGAGTCTGTG	3
NFκB	Chromosome 4<br>103,421,208	Premade PyroMark reference number: PM00051443<br>See Castellano-castillo et al. [1]	7
TNF-α	Chromosome 6<br>31,543,507	F: GGAAAGGATATTATGAGTATTGAAAGTATG<br>R: ACACTCACCTCTTCCCTCTAA<br>S1: ATTATGAGTATTGAAAGTATGAT	5
VDR	Chromosome 12<br>48,297,772	Premade PyroMark reference number: PM00110908<br>See Castellano-castillo et al. [1]	6
