# Supplementary material for: Association between variation of circulating 25-OH vitamin D and methylation of secreted frizzled-related protein 2 in colorectal cancer
Source: Clin Epigenetics. 2020 Jun 9;12:83. doi: 10.1186/s13148-020-00875-9 (PMC7285750; doi:10.1186/s13148-020-00875-9)
Supplement: Supplementary file 6 — Additional file 6: Table S3. General data, clinical and biochemical variables of control and CRC patients. [file 13148_2020_875_MOESM6_ESM.pdf]

Table S3. General data, clinical and biochemical variables of control and CRC patients.

Data are expressed as mean ± standard deviations or percentage. Asterisk indicates significant difference according to Chi squared test for variables expressed as percentage (*p*<0.05) and Welch´s two test for parametric variables (<0.05) **Abbreviations**: CRC: Colorectal cancer; BMI: Body mass index; HDL-c: High density lipoprotein cholesterol; LDL-c: Low density lipoprotein cholesterol.

Variables	Control (116)	CRC (85)
Age (years)	50.14 ± 14.69	67.74 ± 10.53*
Sex (female/male) (%)	57.32/42.68	28.24/71.76*
BMI (kg/m^2)	31.81 ± 10.45	27.21 ± 4.00*
Glucose (mg/dl)	103.19 ± 26.28	123.69 ± 54.63*
Insulin (μUI/ml)	12.23 ± 9.82	6.28 ± 4.94*
Triglycerides (mg/dl)	124.92 ± 59.62	161.54 ± 78.50*
Total cholesterol (mg/dl)	201.88 ± 36.91	172.94 ± 40.77*
HDL-c (mg/dl)	51.90 ± 13.33	41.40 ± 13.90*
LDL-c (mg/dl)	125.17 ± 30.15	103.34 ± 33.20*
